# Polyamines enhance repeat-associated non-AUG translation from CCUG repeats by stabilizing the tertiary structure of RNA

**DOI:** 10.1016/j.jbc.2025.108251

**Published:** 2025-01-31

**Authors:** Akihiro Oguro, Takeshi Uemura, Kodai Machida, Kanta Kitajiri, Ayasa Tajima, Takemitsu Furuchi, Gota Kawai, Hiroaki Imataka

**Affiliations:** 1 Division of Physical Fitness, Department of Molecular Physiology, The Jikei University School of Medicine, Tokyo, Japan; 2 Faculty of Pharmacy and Pharmaceutical Sciences, Josai University, Saitama, Japan; 3 Department of Applied Chemistry, Graduate School of Engineering, University of Hyogo, Himeji, Japan; 4 Department of Molecular Biology, The Jikei University School of Medicine, Tokyo, Japan; 5 Department of Life Science, Faculty of Advanced Engineering, Chiba Institute of Technology, Chiba, Japan

**Keywords:** polyamine, RAN translation, repeat expansion disorder, cell-free protein synthesis system, spermine, RNA structure, neurodegenerative disease

## Abstract

Repeat expansion disorders are caused by abnormal expansion of microsatellite repeats. Repeat-associated non-AUG (RAN) translation is one of the pathogenic mechanisms underlying repeat expansion disorders, but the exact molecular mechanism underlying RAN translation remains unclear. Polyamines are ubiquitous biogenic amines that are essential for cell proliferation and cellular functions. They are predominantly found in cells in complexes with RNA and influence many cellular events, but the relationship between polyamines and RAN translation is yet to be explored. Here, we show that, in both a cell-free protein synthesis system and cell culture, polyamines promote RAN translation of RNA-containing CCUG repeats. The CCUG-dependent RAN translation is suppressed when cells are depleted of polyamines but can be recovered by the addition of polyamines. Thermal stability analysis revealed that the tertiary structure of the CCUG-repeat RNA is stabilized by the polyamines. Spermine was the most effective polyamine for stabilizing CCUG-repeat RNA and enhancing RAN translation. These results suggest that polyamines, particularly spermine, modulate RAN translation of CCUG-repeat RNA by stabilizing the tertiary structure of the repeat RNA.

Repeat expansion disorders are a group of intractable diseases caused by abnormal expansion of microsatellite repeats (1, 2). Microsatellite repeats are various repeated units of 2 to 12 base pairs within a gene. More than 50 human diseases have been reported to be caused by the expansion of microsatellite repeats in various causative genes, most of which are responsible for a wide range of neuromuscular diseases. Expanded repeats can be found throughout a gene, including not only the coding regions, but also the non-coding regions, such as promoters, 5′-untranslated regions (UTRs), introns, or 3′-UTRs. Repeat expansion in coding regions results in the abnormal insertion of additional amino acids into the translated protein. Three types of expanded repeats in the coding region have been reported so far: CAG, GCN, and GGC repeats, which code for polyglutamine, polyalanine, and polyglycine, respectively (3, 4, 5, 6). These stretches can form β-sheets, resulting in insoluble aggregates and leading to either gain or loss of function at the protein level, termed protein toxicity. In contrast, repeat expansion in non-coding regions leads to dysfunction at the RNA level, termed RNA toxicity. RNAs with expanded repeats sequester RNA-binding proteins and form RNA-protein aggregates that can be observed in the nucleus as RNA foci, resulting in cytotoxicity. In some cases, RNA splicing can be inhibited by the sequestration of splicing factors by repeat RNAs.

Another pathogenic mechanism, repeat-associated non-AUG (RAN) translation, was proposed recently (7, 8, 9). RAN translation is a non-canonical translation that depends on some extended repeats without the AUG initiation codon. It was first reported in 2011 and has now been found in seven different repeat sequences in non-coding regions, and one repeat in coding region (10). The RAN translation products form aggregates, which can be toxic in cultured cells and in model organisms (7, 11). The mechanism underlying RAN translation remains unclear, but the tertiary structure of the repeat RNA may play a critical role in the expression of RAN translation (9, 12, 13, 14). RAN translation from CCTG repeat expansion in intron one of *CNBP* was reported in 2017 (15). It has been known that CCTG repeat expansion in *CNBP* causes myotonic dystrophy type 2 (DM2), which is a complex disorder characterized by myotonia and muscle dysfunction (16). RAN translation products from CCUG repeats have been found as aggregates in human brains at autopsy and were reported to be cytotoxic in cultured cells (15). Importantly, the CCUG repeat folds into a tight structure (17, 18).

Polyamines are biogenic amines that are essential for cell proliferation and cellular functions and are mainly found in cells in complexes with RNA (19). Putrescine (Put), spermidine (Spd) and spermine (Spm) are the most abundant polyamines in mammalian cells. Put is synthesized from ornithine by ornithine decarboxylase (ODC), the key enzyme in polyamine biosynthesis, and sequentially converted to Spd and Spm by spermidine synthase (SpdSyn) and spermine synthase (SpmSyn), respectively, through the addition of an aminopropyl group from decarboxylated *S*-adenosylmethionine (dcSAM) (Fig. 1) (20). Polyamines interact with a range of biomolecular components, not only RNAs, but also DNAs, proteins, and phospholipids, and influence many molecular events in gene expression, including translation (21). Under physiological conditions, polyamines are fully protonated, which enables them to form ionic bonds with the negative charges on the phosphate backbone of RNA. In addition, they can bind to specific RNA structures through various non-covalent interactions and are known to regulate RNA functions and subsequent translation (22, 23, 24, 25, 26). Therefore, it is imperative to determine whether polyamines can modulate RAN translation. In this study, we examined the effect of polyamines on RAN translation of CCUG-repeat RNA in the HeLa cell extract-derived cell-free protein synthesis (CFPS) system and cultured cells. Furthermore, we investigated the effect of polyamines on the structure of CCUG-repeat RNA through an analysis of the melting temperature (*T*_m_).

**Figure 1:**
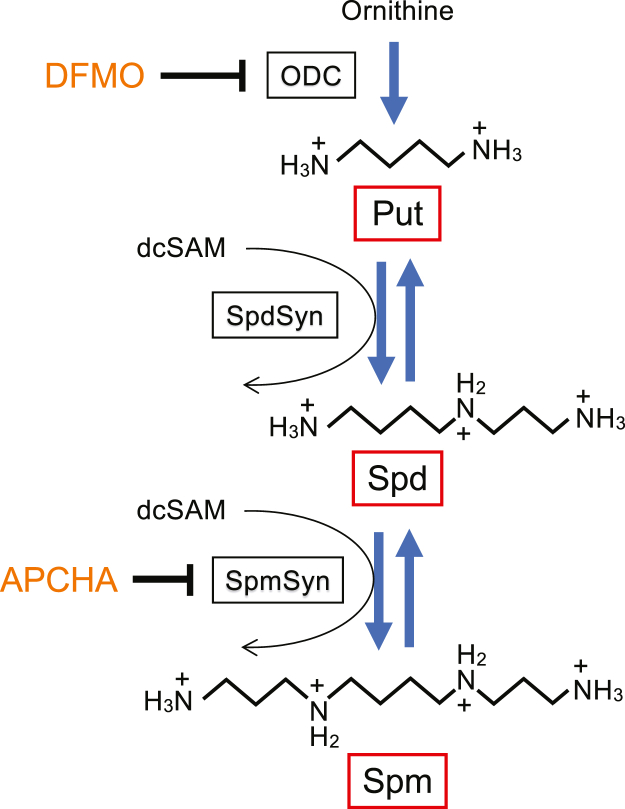
**Polyamine metabolism.** Putrescine (Put) is synthesized from ornithine by ornithine decarboxylase (ODC) and then converted to spermidine (Spd) and spermine (Spm) by spermidine synthase (SpdSyn) and spermine synthase (SpmSyn), respectively. The aminopropyl group required for the synthesis of Spd and Spm is derived from decarboxylated *S*-adenosylmethionine (dcSAM). DFMO is an inhibitor of ODC. APCHA is an inhibitor of SpmSyn.

## Results

### RAN translation from CCUG repeats in the CFPS system

**Figure 2:**
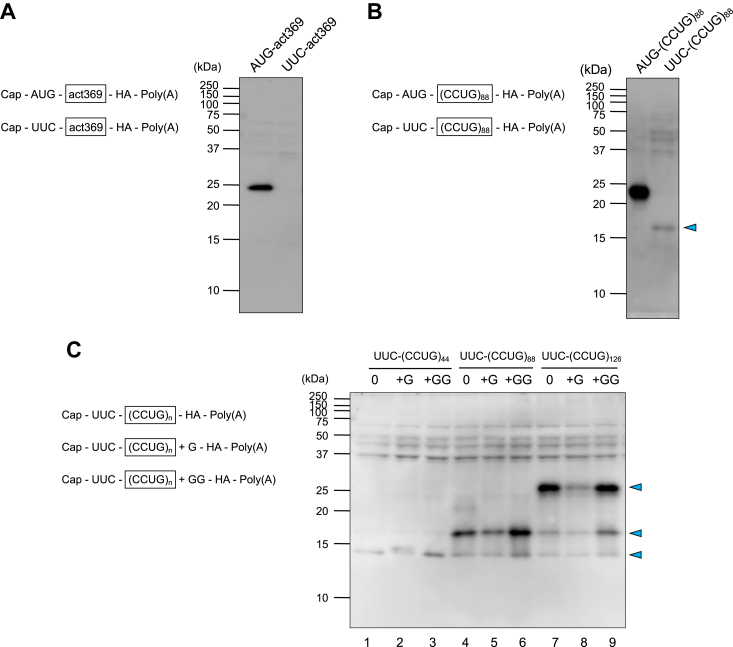
**RAN translation from CCUG-repeat RNAs in the CFPS system.***A*, AUG-act369 and UUC-act369 RNAs were incubated in the CFPS system and the translation products were detected by Western blotting with the anti-HA antibody. *B*, translation products from AUG-(CCUG)_88_ and UUC-(CCUG)_88_ RNAs were detected by Western blotting with the anti-HA antibody. For AUG-(CCUG)_88_, the sample was loaded at 1/10 volume. The arrowhead indicates the RAN translation product. *C*, RAN translation products of three ORFs from UUC-(CCUG)_44_, UUC-(CCUG)_88_ and UUC-(CCUG)_126_ RNAs. Arrowheads indicate RAN translation products.

To examine whether RAN translation is reproducible *in vitro*, capped mRNAs with or without CCUG repeats were translated in the HeLa cell extract-derived CFPS system. The RNA with CCUG repeats [AUG-(CCUG)_88_], in which the CCUG sequence repeats 88 times, comprised the cap-structure, 5′-UTR, initiator AUG, CCUG repeats, intervening sequence, coding sequence of the triple HA-tag, translation terminator UAG, 3′-UTR, and poly(A)_95_ in the 5′ to 3′ direction. The RNA without the repeat sequence (AUG-act369) possessed a portion of β-actin (nucleotides 4–372 of the β-actin ORF, designated act369) in place of the CCUG repeats. Non-AUG RNAs [UUC-(CCUG)_88_ and UUC-act369] contained the UUC codon instead of the initiator AUG (Fig. S1). These RNAs were transcribed *in vitro* (Fig. S2) and incubated in the CFPS system. The translation products were detected by Western blotting with an anti-HA antibody. A protein of the expected size was synthesized from AUG-act369, but no protein was appreciably synthesized from UUC-act369, indicating that translation started at the initiator AUG (Fig. 2*A*). Similarly, a 23-kDa product was synthesized from AUG-(CCUG)_88_ but not from UUC-(CCUG)_88_ (Fig. 2*B*). Instead, translation of the UUC-(CCUG)_88_ RNA yielded one smaller (approximately 16 kDa) product, most likely a RAN translation product.

To confirm that this product is a RAN translation product, we tested UUC-(CCUG)_88_ RNAs with one (G) and two nucleotides (GG) inserted between the CCTG repeat sequence and the HA-tag coding sequence such that +1 and +2 frames, respectively, led to the HA-tag sequence in each RNA (Figs. 2*C*, S1, and S2), as the RAN translation has been reported to occur in all three frames (15). Western blotting showed that the 16 kDa product was synthesized from the +1 (+G) and +2 (+GG) frames, as well as the original frame (0 frame) (Fig. 2*C*, lanes 4–6). Furthermore, UUC-(CCUG)_44_ and UUC-(CCUG)_126_ RNAs, in which the CCUG unit repeats 44 and 126 times, respectively, were translated in the CFPS system (Fig. 2*C*, lanes 1–3 and 7–9). From the UUC-(CCUG)_126_ RNAs, longer (approximately 26 kDa) products were synthesized in amounts comparable to those for each UUC-(CCUG)_88_ RNA in all the frames (0, +G, and +GG). Remarkably, translation with RNAs containing a smaller number of repeats [UUC-(CCUG)_44_ RNAs] yielded a small amount of product (Figs. 2*C* and S3), supporting the idea that the products from the UUC-(CCUG)_88_ and (CCUG)_126_ RNAs are synthesized through RAN translation. These results validate the use of the HeLa cell extract-derived CFPS system to study the mechanism underlying RAN translation.

### Effects of polyamines on RAN translation in the CFPS system

**Figure 3:**
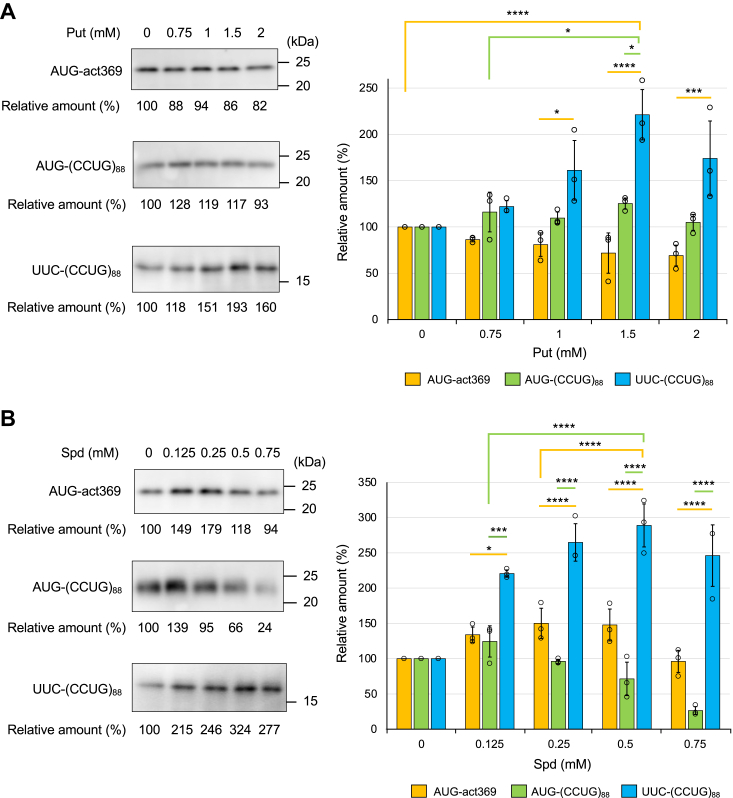

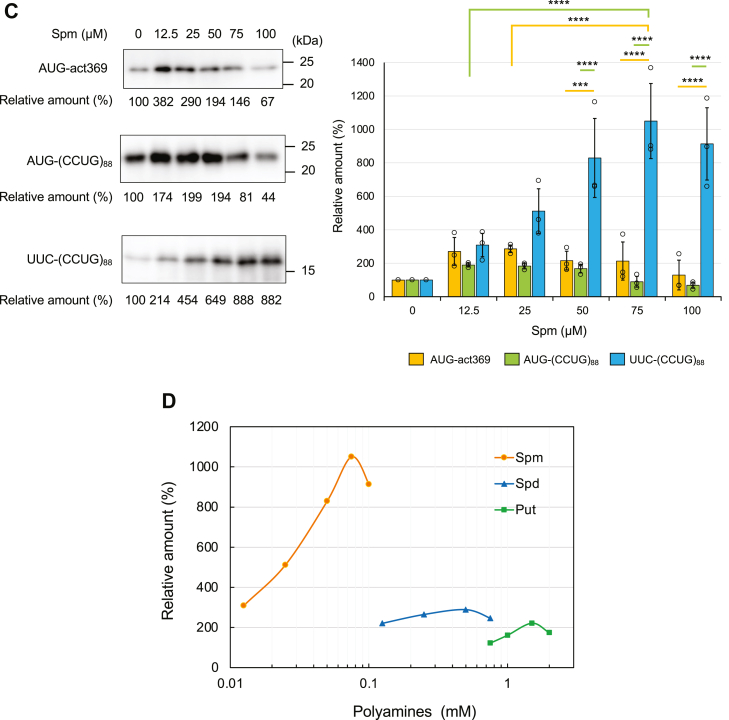
**Effects of polyamines on RAN translation in the CFPS system.***A*–*C*, AUG-act369, AUG-(CCUG)_88,_ and UUC-(CCUG)_88_ RNAs were translated with different concentrations of Put (*A*), Spd (*B*), and Spm (*C*) in the CFPS system, and products were detected by Western blotting with the anti-HA antibody. Relative amounts of the translation products were calculated from the intensity of each protein band with the 0 mM (μM) polyamine control set to 100%. Values are presented as means ± SD (n = 3). ∗*p* < 0.05, ∗∗∗*p* < 0.005, ∗∗∗∗*p* < 0.001. Each data point is represented by an open circle (*A*–*C*). *D*, relative amounts of the RAN products as a function of Put, Spd and Spm concentration are displayed on a semi-log graph. Each point represents the mean (*A*–*C*).

The basal levels of Put, Spd, and Spm in the CFPS system were determined experimentally to be 12.4 ± 4.0 μM, 24.7 ± 0.6 μM, and 39.1 ± 1.9 μM [mean ± standard deviation (SD) (n = 3)], respectively. To determine the effect of polyamines on RAN translation, different concentrations of Put, Spd, or Spm were added to the basal levels. The translation products from UUC-(CCUG)_88_, AUG-(CCUG)_88_, and AUG-act369 RNAs were analyzed by Western blotting using the anti-HA antibody (Fig. 3, *A*–*C*). The addition of each polyamine significantly promoted the RAN translation of UUC-(CCUG)_88_ in a concentration-dependent manner (Fig. 3, *A*–*C*, all data are shown in Fig. S4, *A*–*C*). Spm was prominently effective, requiring much lower doses than Put and Spd for high activity (Fig. 3*D*). RAN translation of each reading frame of (CCUG)_88_-repeat RNA was promoted by the addition of 75 μM Spm (Fig. S5). Although the AUG-derived translation was increased to some extent by the addition of polyamines, this may simply reflect a previous observation that polyamines generally promote protein synthesis, whereas higher levels of polyamines inhibited translation in the HeLa cell extract-derived CFPS system (27). Notably, the highest levels of each polyamine tested in these experiments reduced all translation activity (Fig. 3, *A*–*C*). Thus, polyamines have a specific effect on RAN translation, which differs from the general effect on protein synthesis. Finally, to determine whether there is a direct interaction between polyamines and CCUG-repeat RNA in the CFPS system, mRNAs were purified from the HeLa cell extract supplemented with Spm, and the polyamine bound to the purified RNA was quantified by HPLC. The results showed that Spm is associated with mRNA in the CFPS system (Table S1).

### Effects of polyamines on RAN translation in cultured cells

**Figure 4:**
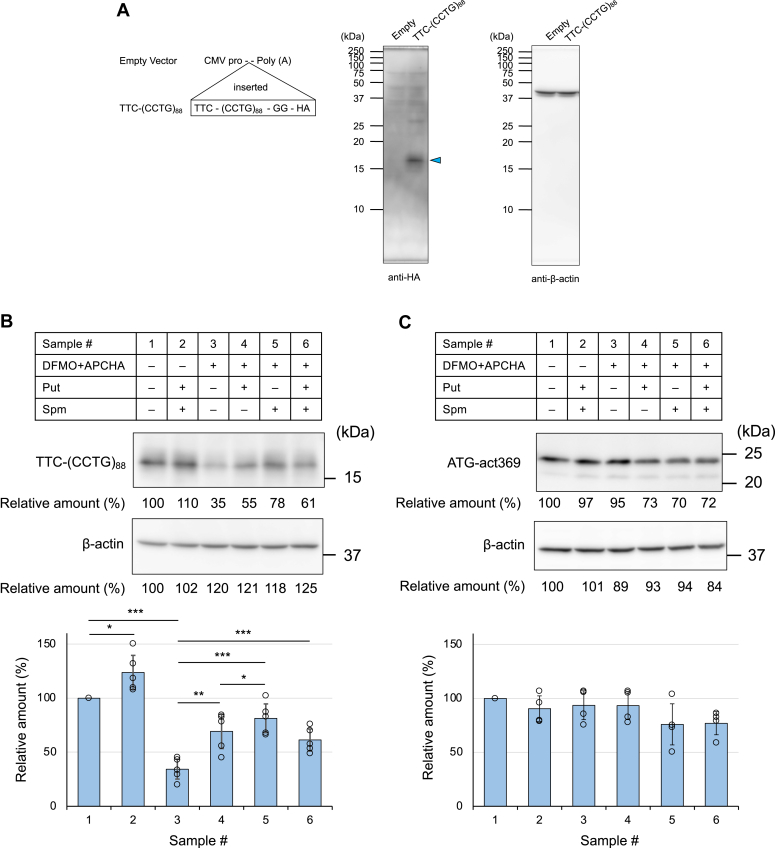

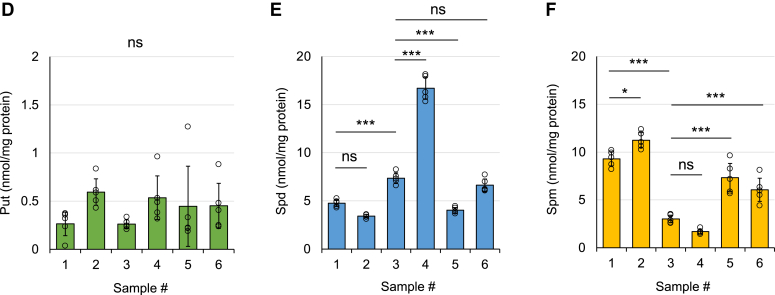
**Effects of polyamines on RAN translation in cells**. *A*, detection of the RAN translation product. The lysates from cells transfected with the empty plasmid or the plasmid containing TTC-(CCTG)_88_ were analyzed by Western blotting with the anti-HA antibody. The arrowhead indicates the RAN translation product (anti-HA blot). Intracellular β-actin was detected as a loading control (anti-β-actin blot). *B*, the RAN translation product in the cells treated with polyamine synthesis inhibitors (DMFO and APCHA) and/or additional polyamines was detected by Western blotting with the anti-HA antibody. Intracellular β-actin was detected as a loading control. Relative amounts of the translation products were calculated from the intensity of each protein band (normalized to the actin control) with sample #1 set to 100%. Values are presented as means ± SD (n = 5). ∗*p* < 0.05, ∗∗*p* < 0.01, ∗∗∗*p* < 0.005. *C*, the translation product from the ATG-act369 construct was analyzed as in (*B*). Values are presented as means ± SD (n = 4). *D*–*F*, cellular concentrations of Put (*D*), Spd (*E*), and Spm (*F*) in each sample. Values are presented as means ± SD (n = 5). ∗*p* < 0.05, ∗∗∗*p* < 0.005, ns: not significant. Each data point is represented by an *open circle* (*B*–*F*).

Next, we examined the effect of polyamines on the RAN translation in cultured cells. HEK293 cells were transfected with the plasmid TTC-(CCTG)_88_ and the cell extract was subjected to Western blotting with the anti-HA antibody (Fig. 4*A*). Synthesis of a polypeptide with a molecular weight similar to that of the RAN translation product in the CFPS system was confirmed upon transfection of the TTC-(CCTG)_88_ plasmid. We varied the cellular concentrations of polyamines by adding Put and Spm to the culture medium or treated cells with a combination of two compounds: α-difluoromethylornithine (DFMO), an ODC inhibitor (28), and *N*-(3-aminopropyl)cyclohexylamine (APCHA), a SpmSyn inhibitor (29), with or without Put and/or Spm (Fig. 4, *B*–*F*; see Figure 1 for the synthetic pathway of each polyamine and action sites of DFMO and APCHA).

The addition of Put and Spm to the medium significantly increased the Spm concentration in the cells with a concomitant increase in RAN translation (samples #1 and #2 in Fig. 4, *B*, and *D*–*F*, all data are shown in Fig. S6). Treatment of the cells with DFMO and APCHA resulted in significant suppression of RAN translation, as well as a significant reduction in the Spm level (samples #1 and #3 in Fig. 4, *B*, and *D*–*F*). When Put was added to the cells that had been treated with these inhibitors, the RAN translation recovered to some extent with accumulation of Spd in the cells (samples #3 and #4 in Fig. 4, *B*, and *D*–*F*). Put was converted to Spd, but not to Spm, as SpmSyn had been inhibited by APCHA. The addition of Spm recovered both the RAN translation and Spm level in the cells (samples #3 and #5 in Fig. 4, *B*, and *D*–*F*). The addition of both Put and Spm to the medium also resulted in the recovery of RAN translation (samples #3 and #6 in Fig. 4*B*). The expression of ATG-act369 protein in cells was not significantly affected by the addition or depletion of polyamines (Fig. 4*C*, all data are shown in Fig. S7). Taken together, these results demonstrate that polyamines, particularly Spm, can modulate the RAN translation of CCUG repeats in cells, similar to the CFPS system. RT-qPCR was also performed on RNAs transcribed from the plasmids transfected into the cells (Fig. S8). The results showed that polyamines had only a small effect on the level of CCUG repeats RNA.

### Importance of the tertiary structure of CCUG repeats for RAN translation

**Figure 5:**
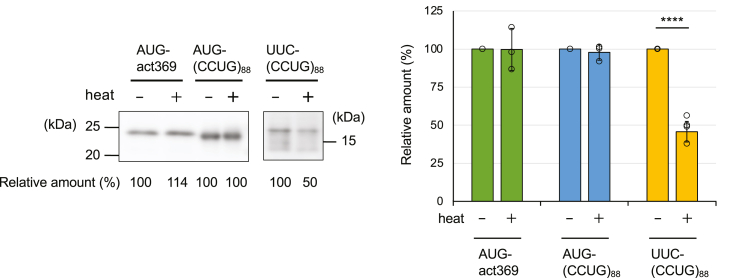
**Effect of heat-denaturation of RNA on translation**. AUG-act369, AUG-(CCUG)_88_, and UUC-(CCUG)_88_ RNAs were heat-denatured (+) or not treated (−) and translated into the CFPS system. Translation products were detected by Western blotting with the anti-HA antibody. Relative amounts of the translation products were calculated from the intensity of each protein band, with the non-denatured control of each RNA set to 100%. Relative amounts of the translation products were calculated from the intensity of each protein band with the value of “heat –” set to 100%. Values are presented as means ± SD [n = 3 for AUG-act369 and AUG-(CCUG)_88_ and n = 4 for UUC-(CCUG)_88_]. ∗∗∗∗*p* < 0.001. Each data point is represented by an open circle.

To understand the mechanism by which polyamines, particularly Spm, modulate RAN translation, we examined the relationship between the tertiary structure of the CCUG-repeat RNA and RAN translation. When the UUC-(CCUG)_88_ RNA was heat-denatured and incubated in the CFPS system, RAN translation was dramatically impaired (Figs. 5 and S9). Heating did not affect the integrity of the CCUG-repeat RNA (Fig. S10). In contrast, translation of AUG-act369 and AUG-(CCUG)_88_ RNA was not affected by heat-denaturation (Figs. 5 and S9): a portion of the heat-denatured CCUG-repeat RNA seems to be renatured during incubation (Fig. S11). These results indicate that the tertiary structure of the RNA is important for the induction of RAN translation.

**Figure 6:**
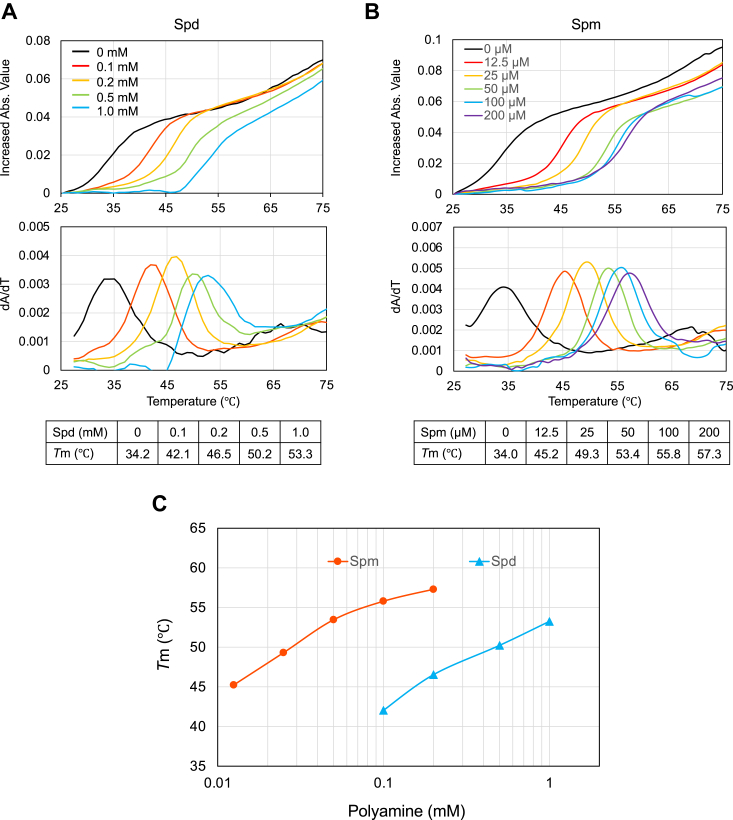
**Effects of polyamines on the tertiary structure of CCUG-repeat RNA.***A* and *B*, The absorbance variation at 260 nm for (CCUG)_44_*versus* temperature in the presence of different concentrations of Spd (*A*) and Spm (*B*) (*upper panels*) and its first derivative curves (*lower panels*). *C*, the *T*_m_ values for each polyamine concentration from the tables are plotted as a function of the concentration of each polyamine on a semi-log graph.

Previous studies have shown that polyamines may stabilize the tertiary structure of RNA (26, 30). Thus, we explored the effect of polyamines on the structure of the CCUG-repeat RNA. The *T*_m_ of the CCUG-repeat RNA was analyzed from UV melting curve, since this is a usual method to study the structural stability of RNA (31). The *T*_m_ of an RNA containing 44 repeats of CCUG increased when the concentration of Spd and Spm was increased (Fig. 6, *A* and *B*), indicating that Spd and Spm stabilize the tertiary structure of the CCUG-repeat RNA. An RNA containing 88 repeats of CCUG, which was used in the RAN translation experiments, appeared to be unsuitable for this experiment, because melting process of a large RNA structure could be rather complex (31). Notably, the effective range of the Spm concentration on the *T*_m_ of the CCUG-repeat RNA was much lower than that of Spd (Fig. 6*C*). These results support the idea that polyamines, particularly Spm, stabilize the tertiary structure of the CCUG-repeat RNA, thereby inducing RAN translation.

### Localization of the initiation codon for CCUG repeats-dependent RAN translation

**Figure 7:**
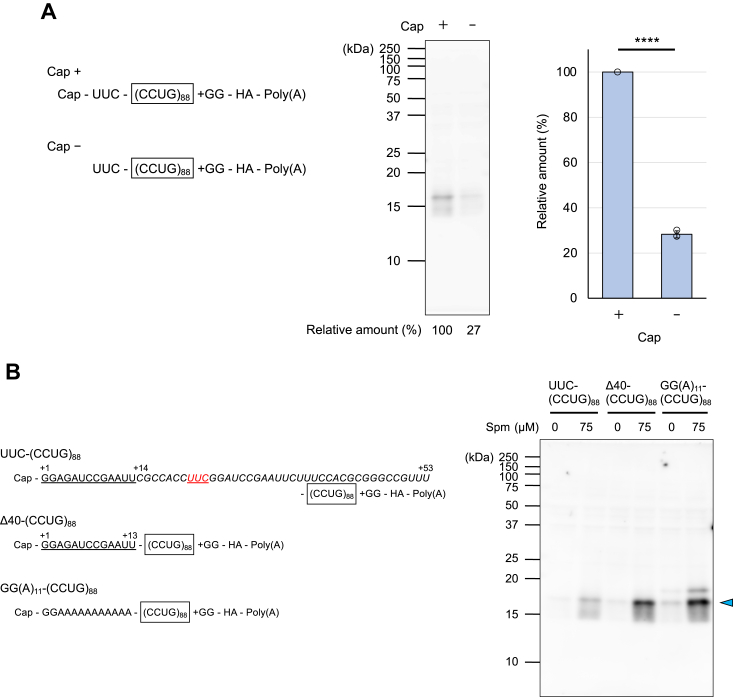
**Effects of the cap structure and upstream sequences of the CCUG repeats on RAN translation.***A*, translation of capped or uncapped UUC-(CCUG)_88_ RNA in the CFPS system (Cap + or Cap –, respectively). Relative amounts of the translation products were calculated from the intensity of each protein band with the value of “Cap +” set to 100%. Values are presented as means ± SD (n = 3). ∗∗∗∗*p* < 0.001. Each data point is represented by an *open circle*. *B*, translation of UUC-, Δ40-, or GG(A)_11_-(CCUG)_88_ RNA in the presence or absence of 75 μM Spm in the CFPS system. Products were detected by Western blotting with the anti-HA antibody. The arrowhead indicates the RAN translation product.

Finally, we attempted to localize the initiation site of the CCUG repeats-dependent RAN translation. Translation from UUC-(CCUG)_88_ was cap-dependent (Figs. 7*A*, S12*A*, and S13), indicating that scanning of the 43S ribosomal preinitiation complex (PIC) should start from the 5′ end of the RNA. However, the idea that the CCUG repeat sequence would act as an IRES cannot be ruled out, although it is highly likely that the ribosomes capture the 5′-end of the message. There is a spacer sequence of 53 nucleotides between the 5′ end and the first CCUG in the repeat tract in the RNA template (Figs. 7*B* and S1), which is derived from the original plasmid but not from the genome sequence, and translation may start at a triplet in this spacer region. To evaluate this possibility, the 40 nucleotides from position +14 to +53 in this spacer sequence were deleted (Figs. 7*B* and S1), and the resulting RNA [Δ40-(CCUG)_88_, Fig. S12*B*] was translated in the CFPS system. A translation product with the same molecular weight as UUC-(CCUG)_88_ was still synthesized from Δ40-(CCUG)_88_ in response to Spm (Figs. 7*B* and S14). To examine whether any codon in the remaining 13 nucleotides (+1 to +13) could serve as the translation start site, this 13-nucleotide sequence, except for the first two nucleotides, was replaced with the A tract (Figs. 7*B* and S1), and the resulting RNA [(GG(A)_11_-(CCUG)_88_, Fig. S12*B*] was translated in the CFPS system. We did not simply delete the 13-nucleotide sequence because placement of the tertiary structure of the CCUG repeat near the 5′ end would disturb the entry of the 43S PIC. The Spm-induced RAN translation product was similarly synthesized from this RNA (Figs. 7*B* and S14). It is very unlikely that any of the triplets contained in the GG(A)_11_ sequence (GGA, GAA and AAA) would serve as the initiation codon because none of them are near-AUG codons (32, 33, 34). Conversely, it is likely that the 43S PIC selects a CUG triplet in the CCUG repeats as the initiation codon because the CUG triplet is the second most frequently used initiation codon in eukaryotic cells (32, 34), and other triplets in the CCUG repeats (CCU, UGC, and GCC) act far less efficiently as initiation codons in the CFPS system (Fig. S15).

## Discussion

**Figure 8:**
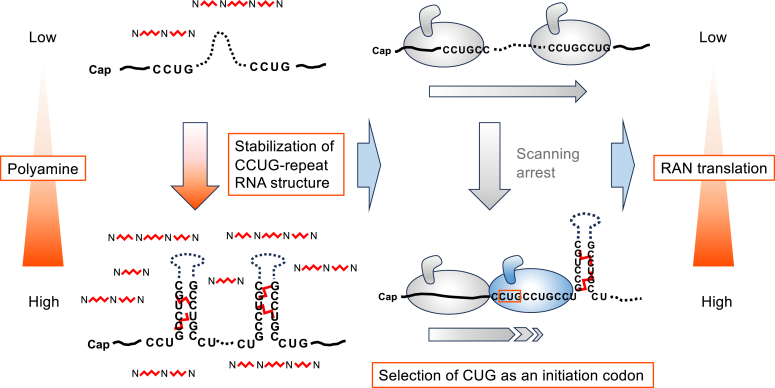
**Molecular model of polyamines-enhanced RAN translation from the CCUG-repeat RNA.** Higher levels of polyamines stabilize the tertiary structure of CCUG-repeat RNA, which eventually inhibits scanning of the 43S PIC. Scanning arrest of the 43S PIC enhances the recognition of a CUG triplet in the CCUG repeats as the initiation codon, which subsequently leads to RAN translation.

We propose a mechanism by which polyamines enhance CCUG repeats-dependent RAN translation (Fig. 8). Polyamines stabilize the tertiary structure of the CCUG-repeat RNA, resulting in scanning arrest of the 43S PIC. Consequently, RAN translation from a CUG codon in the CCUG repeat is enhanced. This model is consistent with previous results showing that overexpression of eIF2A, which affects translation from the non-AUG codon, increases CCUG repeats-dependent RAN translation (35). RAN translation products were detected mainly as a specific band (Figure 2, Figure 3, Figure 4) sometimes with smear-like band patterns (Figs. 5 and 7). We presume that the 43S PIC is arrested just upstream of the tertiary structure of the CCUG-repeat RNA, and thereby the most 5′ CUG triplet in the repeats is selected as the initiation codon with a high probability. This could explain that the length of RAN products between each frame shows little difference (Fig. 2*C*).

It has been reported that polyamines enhance the canonical translation in the CFPS system by increasing the fidelity at the aminoacyl-tRNA binding ribosomes and/or stimulating the assembly of the ribosomal subunit (36). This enhancement by polyamine was also observed in the present study, although the optimal concentrations for the canonical translation differed from those for the RAN translation (Fig. 3, *A*–*C*). Thus, the effect of polyamines on the RAN translation may also include an effect on the canonical translation.

On the other hand, excess polyamines have been reported to reduce the accuracy of codon-anticodon base pair interaction (37). This could be the enhancement of RAN translation by polyamines may be partly due to increased misrecognition of the CUG codon as the initiation codon. However, this may not be the case, because the effect of Spm on CUG-act369 translation was similar to the effect on AUG-act369 translation (Fig. S16). Spm was the most effective polyamine for the enhancement of RAN translation and the stabilization of CCUG-repeat RNA. The chemical properties of this polyamine can account for its superiority; Spm has excess positive charges over Put and Spd, resulting in a higher affinity for some RNAs than others (38, 39, 40), and the longer carbon chain of Spm could effectively stabilize the conformation of the CCUG-repeat RNA.

The findings of this study may contribute to the development of a new therapeutic approach for DM2 caused by repeat expansion. Reducing the Spm level with APCHA could be a promising strategy. Spm-binding RNA, which we previously developed (26), could also be employed to reduce the concentration of free Spm in cells. The next step is to clarify the causal relationship between the polyamine contents and tissue degeneration.

## Experimental procedures

### Plasmid construction

The CCTG repeats were PCR-amplified from pAAV CCTG 1200× (Addgene, #63088) and subcloned into a modified vector based on the pUC-T7-HA poly(A) plasmid (41) for *in vitro* transcription, or into a modified vector based on p3×FLAG-CMV-14 (Sigma-Aldrich) with the HA-tag coding sequence for transfection into cells. Site-directed mutagenesis was performed to construct variants using the KOD-Plus Mutagenesis Kit (Toyobo) according to the manufacturer’s instructions. The integrity of all the plasmids was confirmed by DNA sequencing. For repeat DNA sequences, cycle sequencing reactions were performed in the presence of 1 M betaine. Prior to *in vitro* transcription, template plasmid DNA was linearized using the restriction enzyme *Not* I for the CFPS system or *Xho* I for the *T*_m_ experiments.

### HeLa cell extract-derived CFPS system

*In vitro* translation was performed using the HeLa S3 cell extract–derived CFPS system (27, 42). RNA was transcribed *in vitro* from the *Not* I-digested plasmid with the ARCA cap analog (Jena Bioscience GmbH) using T7 RNA polymerase in a polyamine-free condition supplemented with 25 mM MgCl_2_, followed by purification with the Monarch RNA Cleanup kit (NEB). For uncapped RNAs, ARCA was removed from the mixture. The RNA (0.4 μg) was incubated with the reaction mixture (5 μl), which was supplemented with 0.5 mM Spd (final concentration) as the standard condition or with varying concentrations of polyamine for 1.5 h at 32 °C. Protein products were separated by SDS-PAGE and analyzed by Western blotting. To confirm the integrity of each RNA, RNA samples were electrophoresed on a 1.5% agarose gel with GelGreen (Biotium) under the denaturing conditions provided by the RNA High for Easy Electrophoresis Kit (BioDynamics Laboratory Inc) and detected under blue light. To denature the RNA, samples were heated to 95 °C for 5 min and then immediately placed on ice for 5 min before being incubated with the reaction mixtures.

### Western blot analysis

Proteins resolved by SDS-PAGE were transferred to a PVDF membrane. The membrane was treated with 5% skim milk dissolved in PBS supplemented with Tween 20 (0.1%) or PVDF blocking reagent (Toyobo) at room temperature for 30 to 45 min. Translation products were detected by anti-HA antibody (BioLegend, #901513) as the primary antibody and HRP–labeled anti-mouse IgG antibody (GE Healthcare, #NA931) as the secondary antibody. To detect β-actin as the loading control for the cell lysate, the membrane used for HA-Western blotting was subsequently reacted with HRP-conjugated anti-β-actin antibody (MBL, #PM053-7). Immunoblot images were captured on an LAS 3000mini (GE Healthcare) or Light Capture II (ATTO). Densitometric analysis was performed using ImageJ (43).

### Cell culture and preparation of cell lysate

HEK293 cells were maintained in DMEM with 4.5 g/L glucose (Nacalai or Wako) supplemented with 10% fetal bovine serum (Biosera), 100 units/ml penicillin, and 100 μg/ml streptomycin (Wako). All cells were incubated at 37 °C in a humidified atmosphere of 5% CO_2_. A total of 2 × 10^5^ cells per well were grown in a 6-well plate for 24 h and transfected with 2 μg plasmid DNA using Lipofectamine 3000 Reagent (Invitrogen) according to the manufacturer’s instructions. After 14 h of incubation, the media was changed to fresh media supplemented with aminoguanidine (1 mM, Wako) for all samples and with DFMO (0.5 mM) plus APCHA (0.2 mM) (29), Put (100 μM, Wako), or Spm (50 μM, Nacalai) as appropriate (presented as final concentration in medium). After 48 h incubation, the cells were washed twice with ice-cold D-PBS(−) (Wako) and suspended in RIPA buffer (Nacalai, #16488-34) supplemented with protein inhibitor cocktail (Nacalai, #25955-24). The cells were lysed by repeated sonication and centrifuged at 16,000*g* for 20 min. The protein concentrations of the supernatants were determined using the DC Protein Assay Kit (Bio-Rad).

### Measurement of polyamine contents

Polyamine levels in the HeLa cell extract (for the CFPS system) or cell lysates, and binding to mRNAs were determined by ion-pair HPLC as described previously (44). Briefly, the samples were treated with trichloroacetic acid (5%, final concentration) at 90 °C for 30 min and cooled on ice for 5 min. After centrifuging twice at 21,600*g* for 15 min, the supernatants were collected and analyzed using a Jasco HPLC system with an InertSustain C18 column (GL Science).

### Analysis of T_m_

The *T*_m_ experiments were performed using V-730BIO (JASCO). (CCUG)_44_ RNA was transcribed *in vitro* from the *Xho* I-digested plasmid without the cap analog and dissolved in a buffer (10 mM HEPES-KOH, pH 7.6, 100 mM KCl). The absorbance profiles were recorded at 260 nm from 25 °C to 95 °C at a rate of 1 °C/min.

### Statistical analysis

Statistical analyses were performed using R software, version 4.3 (45). Differences between groups were compared using Tukey’s multiple comparisons test.

## Data availability

All data supporting the findings of this study are available in the paper and its supporting information, or are available from the Corresponding Authors on reasonable request.

## Supporting information

This article contains supporting information (46).

## Conflict of interest

The authors declare that they have no conflicts of interest with the contents of this article.
