# Supplementary material for: Polyamines enhance repeat-associated non-AUG translation from CCUG repeats by stabilizing the tertiary structure of RNA
Source: J Biol Chem. 2025 Jan 31;301(3):108251. doi: 10.1016/j.jbc.2025.108251 (PMC11919584; doi:10.1016/j.jbc.2025.108251)
Supplement: Supporting Information [file mmc1.pdf]

# Supporting Information

**Polyamines enhance repeat-associated non-AUG translation from CCUG repeats by stabilizing the tertiary structure of RNA**

**Oguro *et. al***

**Contents:**

**Supporting Figures (S1-S16)**

**Supporting Table1 (Table S1)**

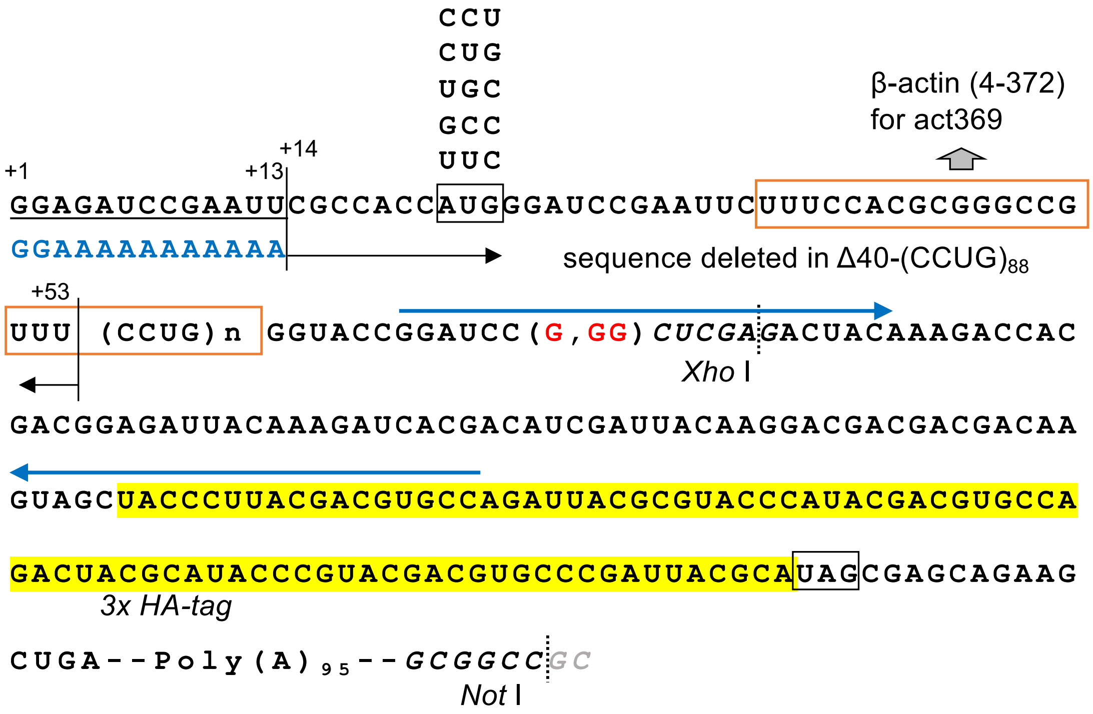


**Figure S1.** RNA sequences used in this study. Nucleotides are numbered from the transcription start site (+1). The CCUG repeat tract is represented by (CCUG)n. The initiation (AUG) and termination (UAG) codons are enclosed by black boxes. Codons replacing the AUG codon are shown above the AUG codon. For the act369 construct, the sequence enclosed by the orange box is replaced by the sequence of truncated β-actin (4-372). The sequence for the triple HA-tag (3× HA-tag) is marked in yellow. The sequence GG(A)$_{11}$ of the GG(A)$_{11}$-(CCUG)$_{88}$ RNA is shown in blue under the original sequence. The *Not* I or *Xho* I site was used to linearize the plasmid for *in vitro* transcription. (G, GG in red): addition of G or GG at this position to provide different ORFs. G is inserted at this position for AUG-(CCUG)$_{88}$ to adjust the reading frames. Blue arrows: primers for RT-qPCR.

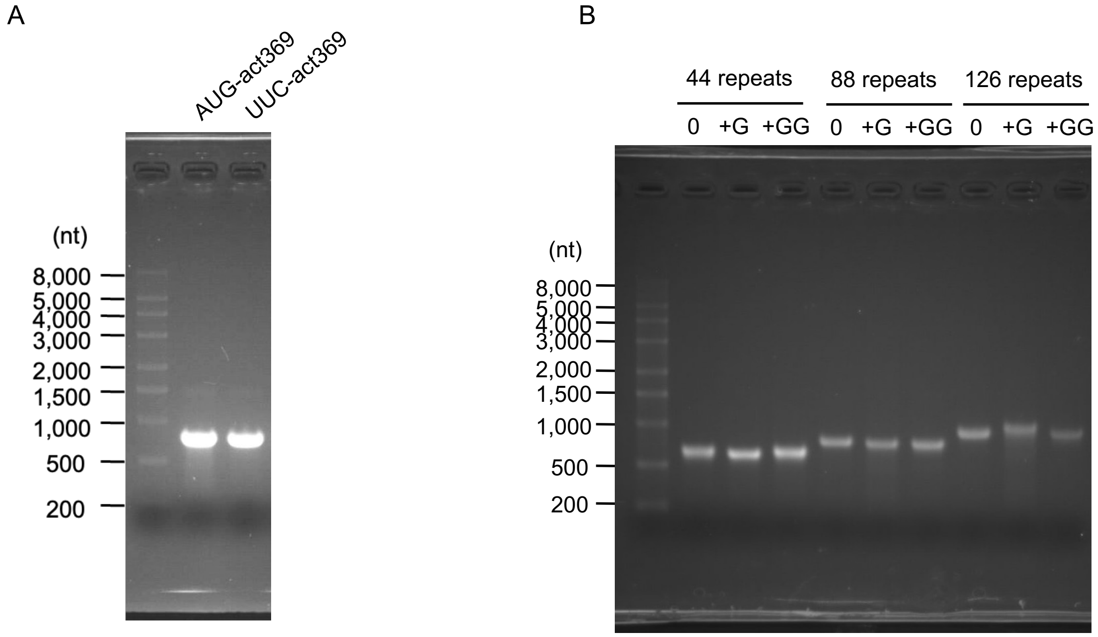


**Figure S2.** *In vitro* transcribed RNAs for the CFPS system. (A) Transcribed AUG-act369 and UUC-act369 were used for the translation in the CFPS system in Figure 2A. (B) Transcribed UUC-$(CCUG)_{44}$, -$(CCUG)_{88}$, and -$(CCUG)_{126}$ RNAs (44 repeats, 88 repeats, and 126 repeats, respectively) in the absence (0), or presence of one or two additional nucleotides (+G or +GG, respectively) were used for the translation in the CFPS system in Figure 2B and 2C.

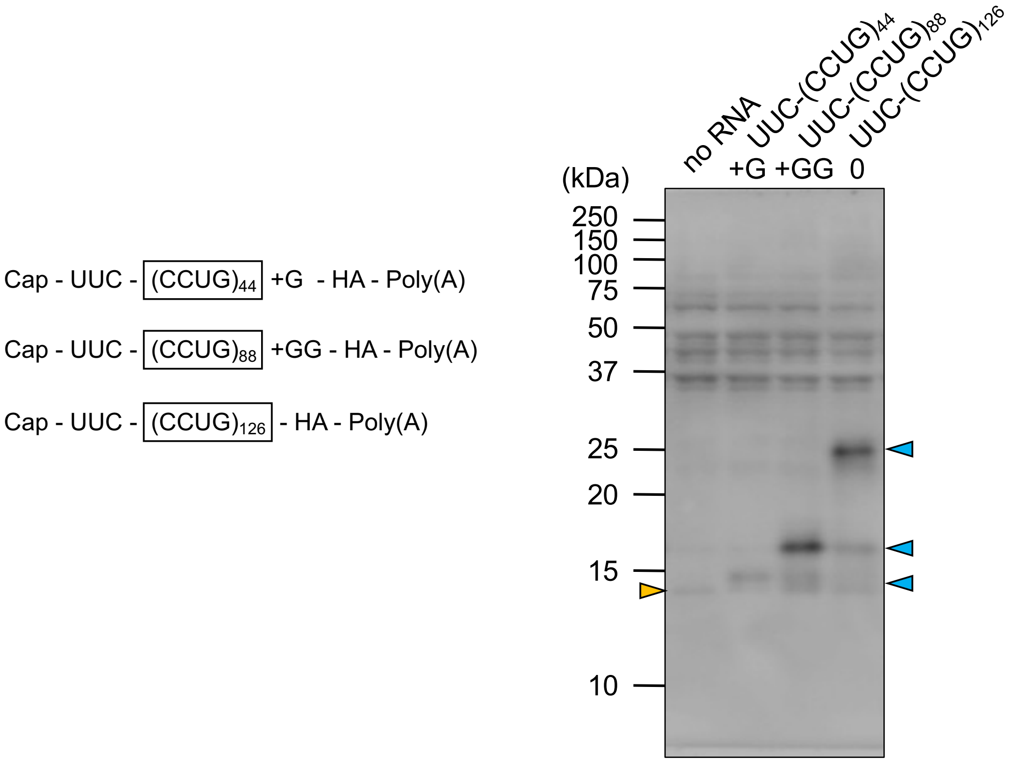


**Figure S3.** RAN translation from UUC-$(CCUG)_{44}$, UUC-$(CCUG)_{88}$, and UUC-$(CCUG)_{126}$ RNAs. Translation products were detected by Western blotting with the anti-HA antibody. Translation from each RNA with the particular reading frame that caused RAN most efficiently in Figure 2C is compared. Blue arrowheads indicate the RAN translation products. An orange arrowhead indicates a non-specific band from the HeLa cell extract.

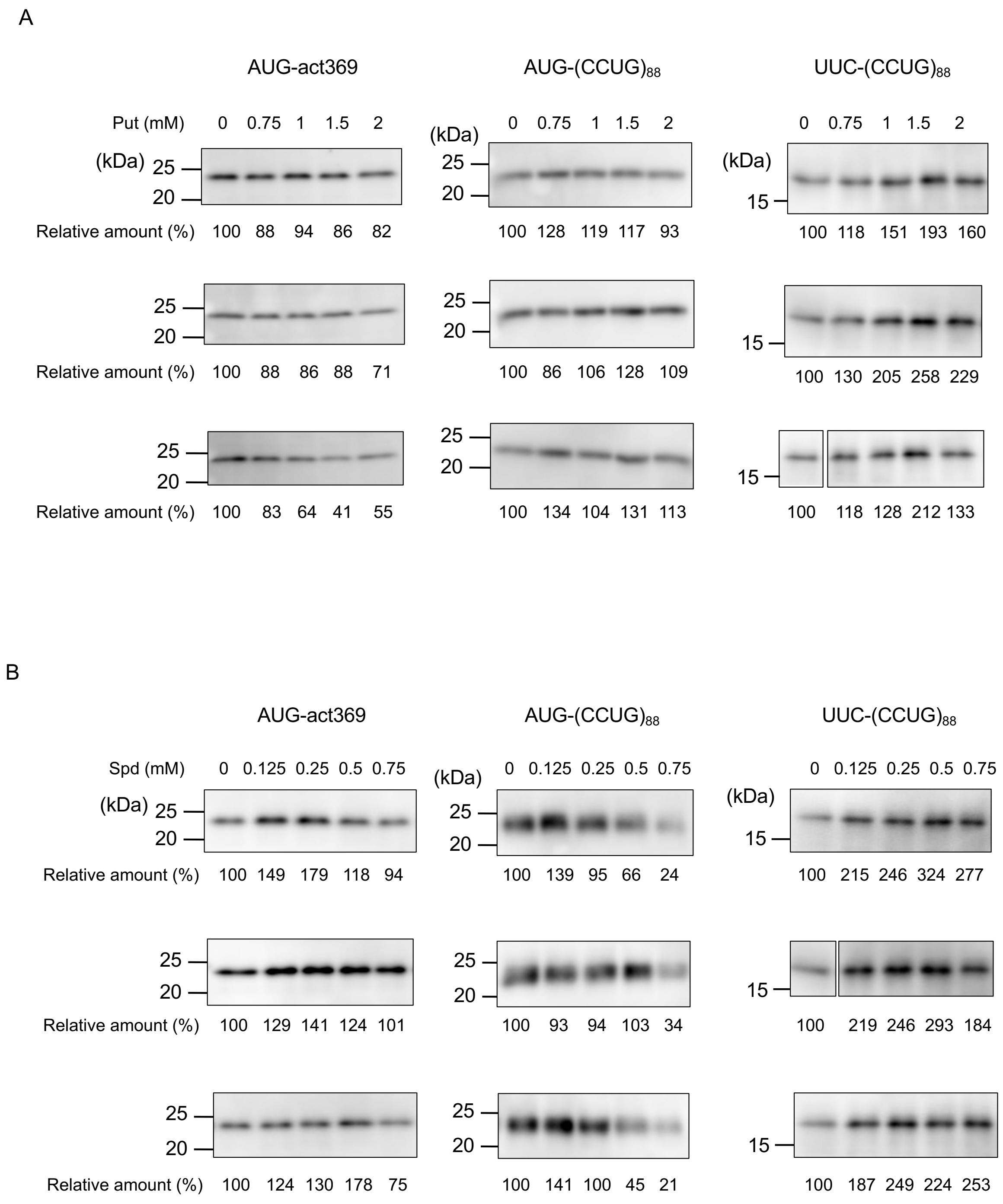
A
AUG-act369
AUG-(CCUG)88
UUC-(CCUG)88
Put (mM) 0 0.75 1 1.5 2
(kDa)
25
20
15
Relative amount (%) 100 88 94 86 82
100 128 119 117 93
100 118 151 193 160
Relative amount (%) 100 88 86 88 71
100 86 106 128 109
100 130 205 258 229
Relative amount (%) 100 83 64 41 55
100 134 104 131 113
100 118 128 212 133
B
AUG-act369
AUG-(CCUG)88
UUC-(CCUG)88
Spd (mM) 0 0.125 0.25 0.5 0.75
Relative amount (%) 100 149 179 118 94
100 139 95 66 24
100 215 246 324 277
Relative amount (%) 100 129 141 124 101
100 93 94 103 34
100 219 246 293 184
Relative amount (%) 100 124 130 178 75
100 141 100 45 21
100 187 249 224 253

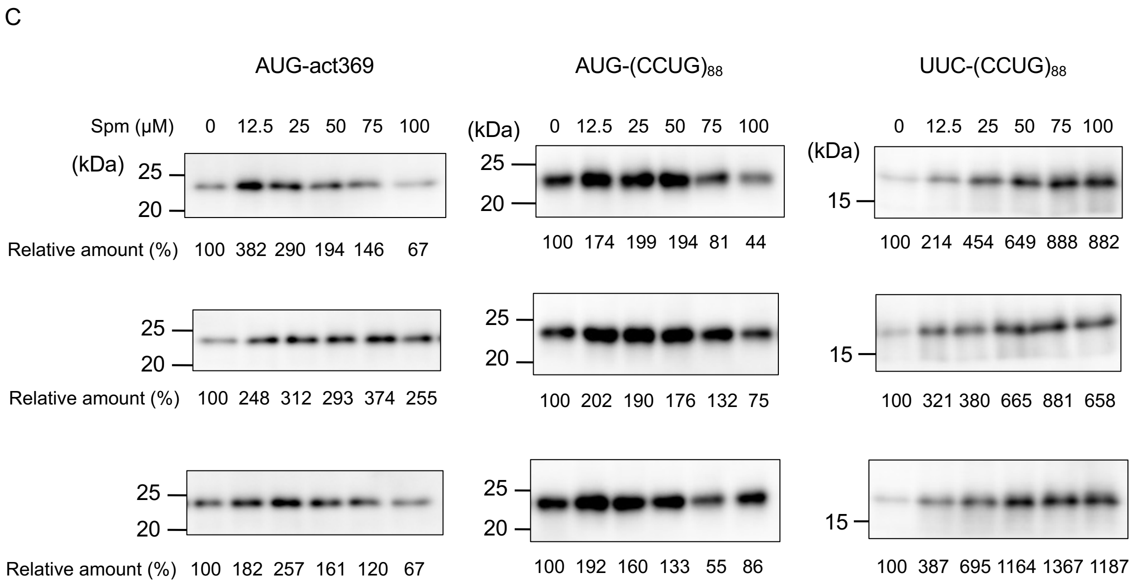


**Figure S4.** Individual data of translation for Figures 3A-3C.

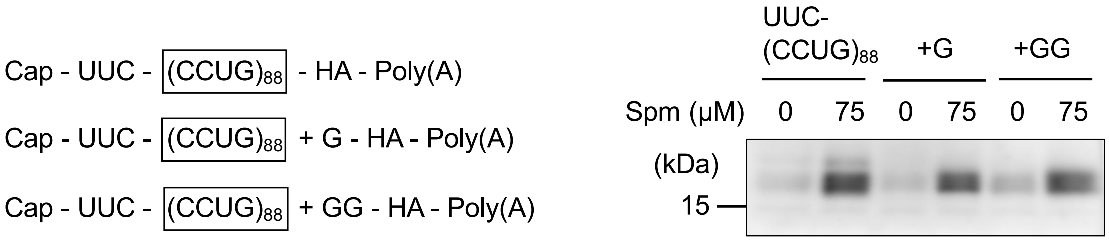


**Figure S5.** Effects of Spm on RAN translation of each reading frame of the $(CCUG)_{88}$-repeat in the CFPS system.

A

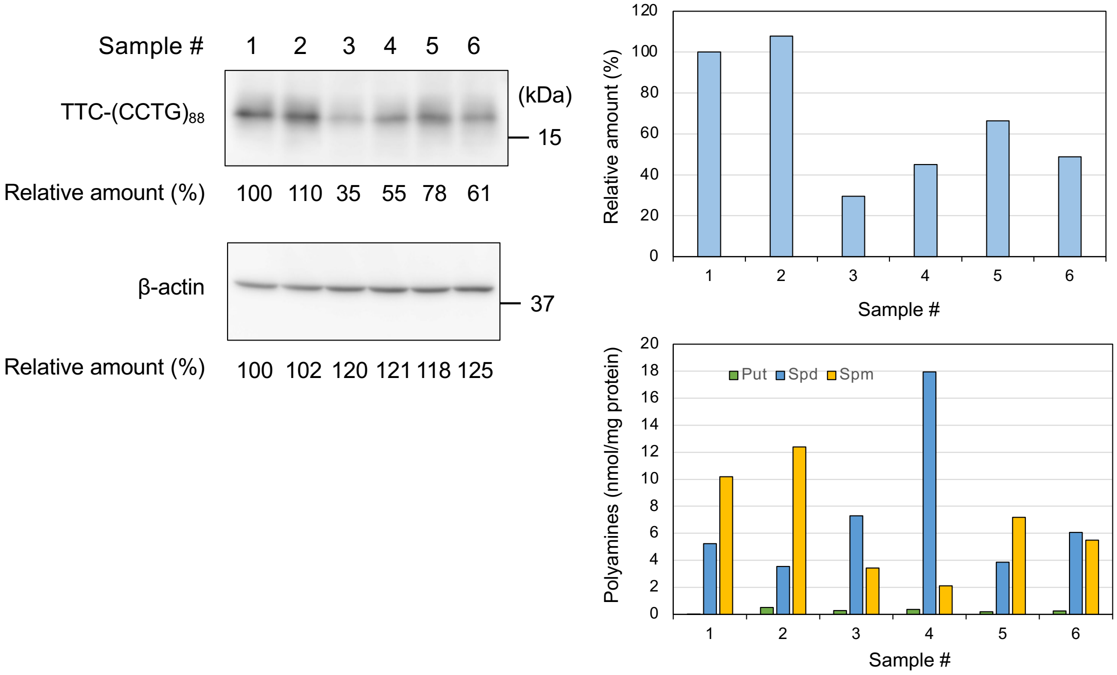
Sample # 1 2 3 4 5 6
TTC-(CCTG)88
(kDa)
15
Relative amount (%) 100 110 35 55 78 61
β-actin
37
Relative amount (%) 100 102 120 121 118 125
Relative amount (%)
Sample #
Put Spd Spm
Polyamines (nmol/mg protein)
Sample #

B

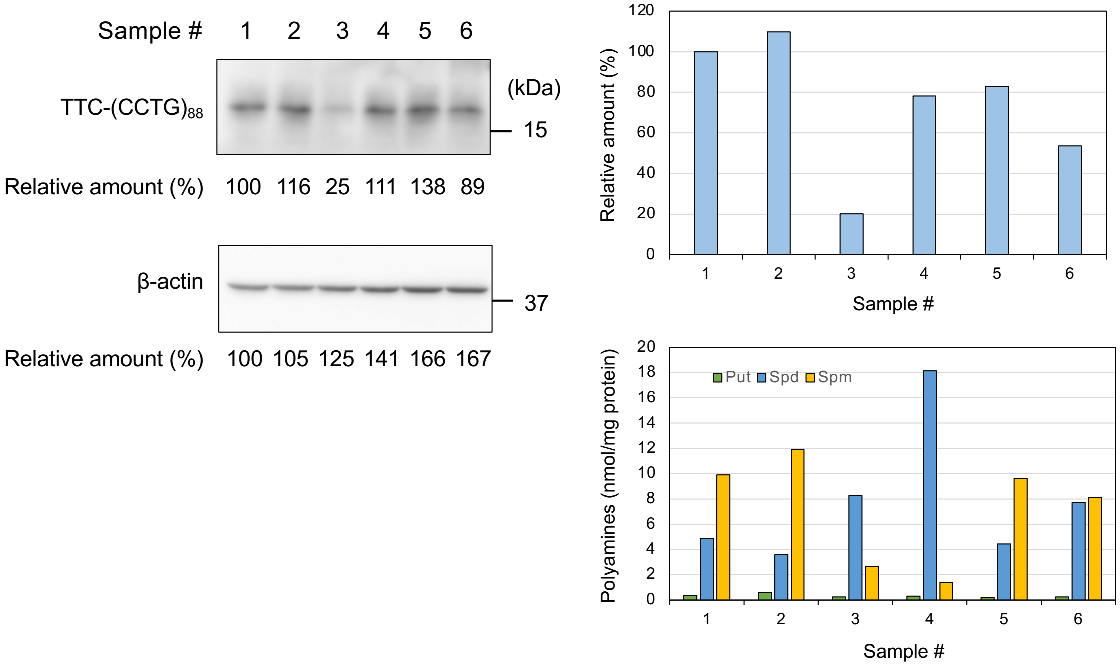
Sample # 1 2 3 4 5 6
TTC-(CCTG)88
(kDa)
15
Relative amount (%) 100 116 25 111 138 89
β-actin
37
Relative amount (%) 100 105 125 141 166 167
Relative amount (%)
Sample #
Put Spd Spm
Polyamines (nmol/mg protein)
Sample #

C

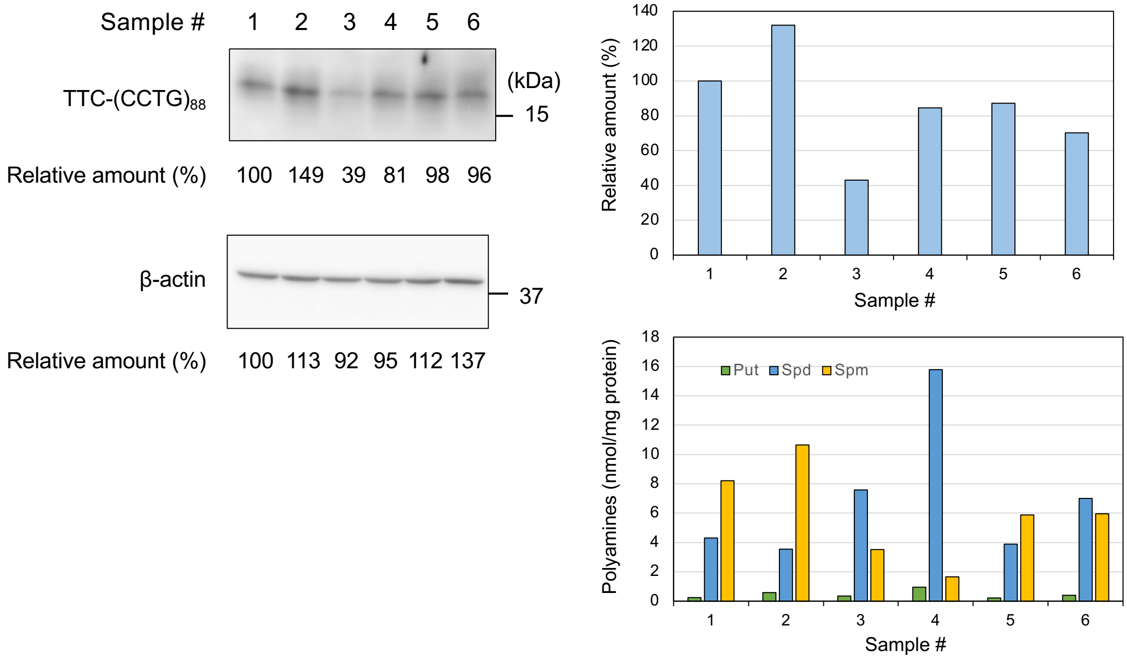
Sample # 1 2 3 4 5 6
TTC-(CCTG)88
(kDa)
15
Relative amount (%) 100 149 39 81 98 96
β-actin
37
Relative amount (%) 100 113 92 95 112 137
Relative amount (%)
Sample #
Polyamines (nmol/mg protein)
Put Spd Spm
Sample #

D

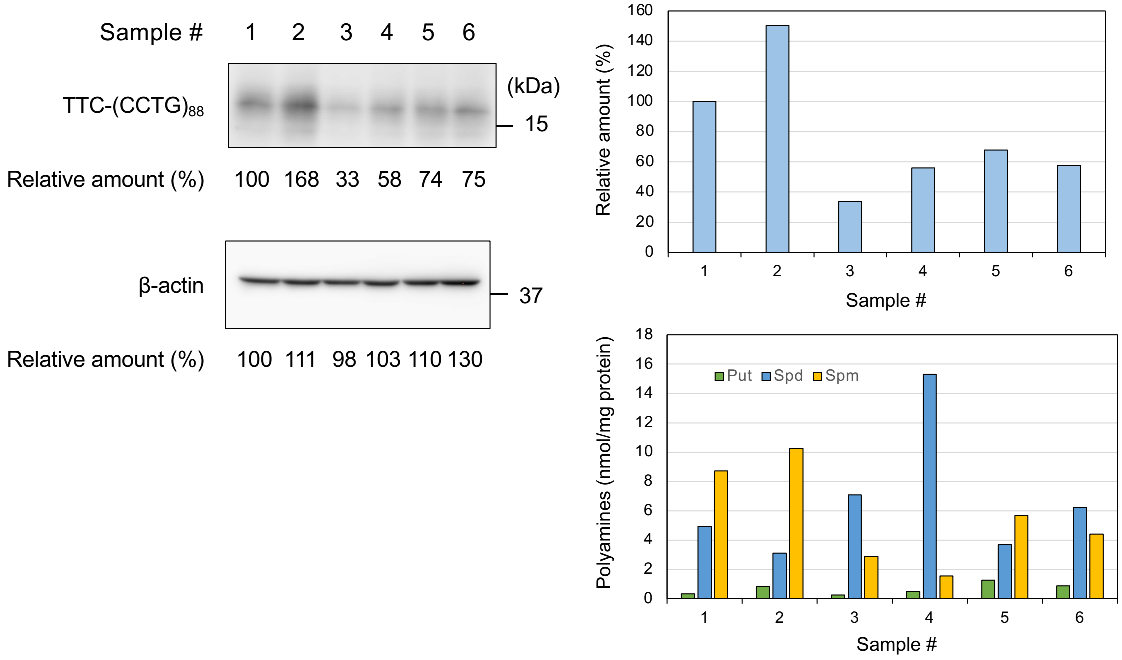
Sample # 1 2 3 4 5 6
TTC-(CCTG)88
(kDa)
15
Relative amount (%) 100 168 33 58 74 75
β-actin
37
Relative amount (%) 100 111 98 103 110 130
Relative amount (%)
Sample #
Polyamines (nmol/mg protein)
Put Spd Spm
Sample #

E

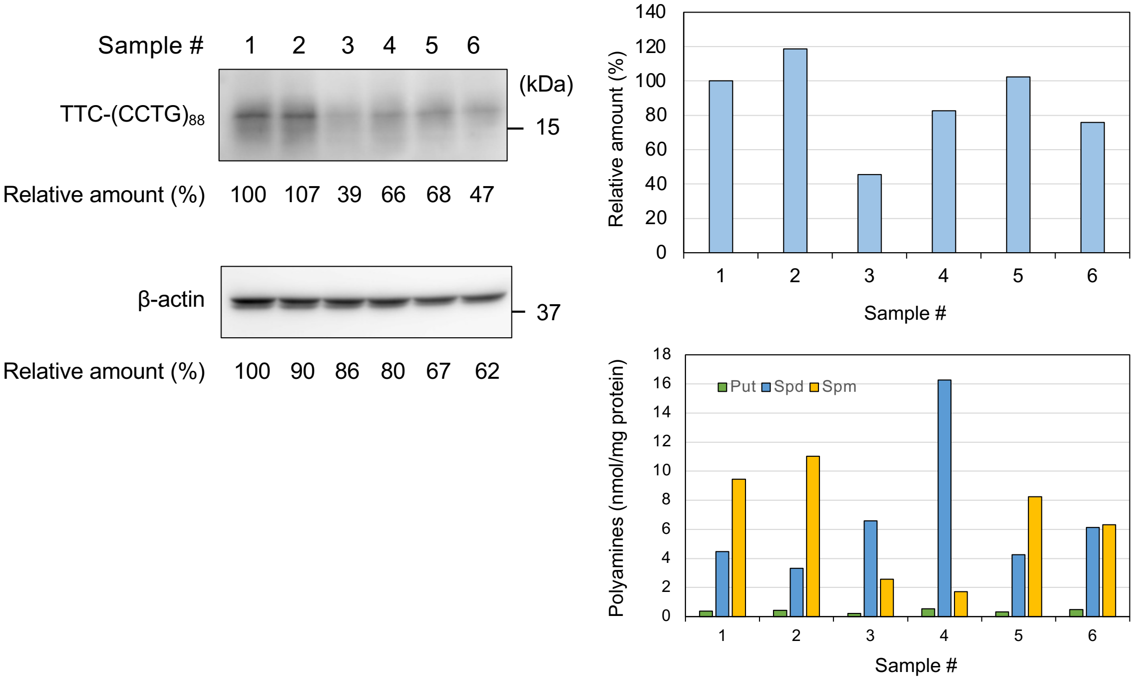


**Figure S6.** Individual data for Figures 4B and 4D-4F.

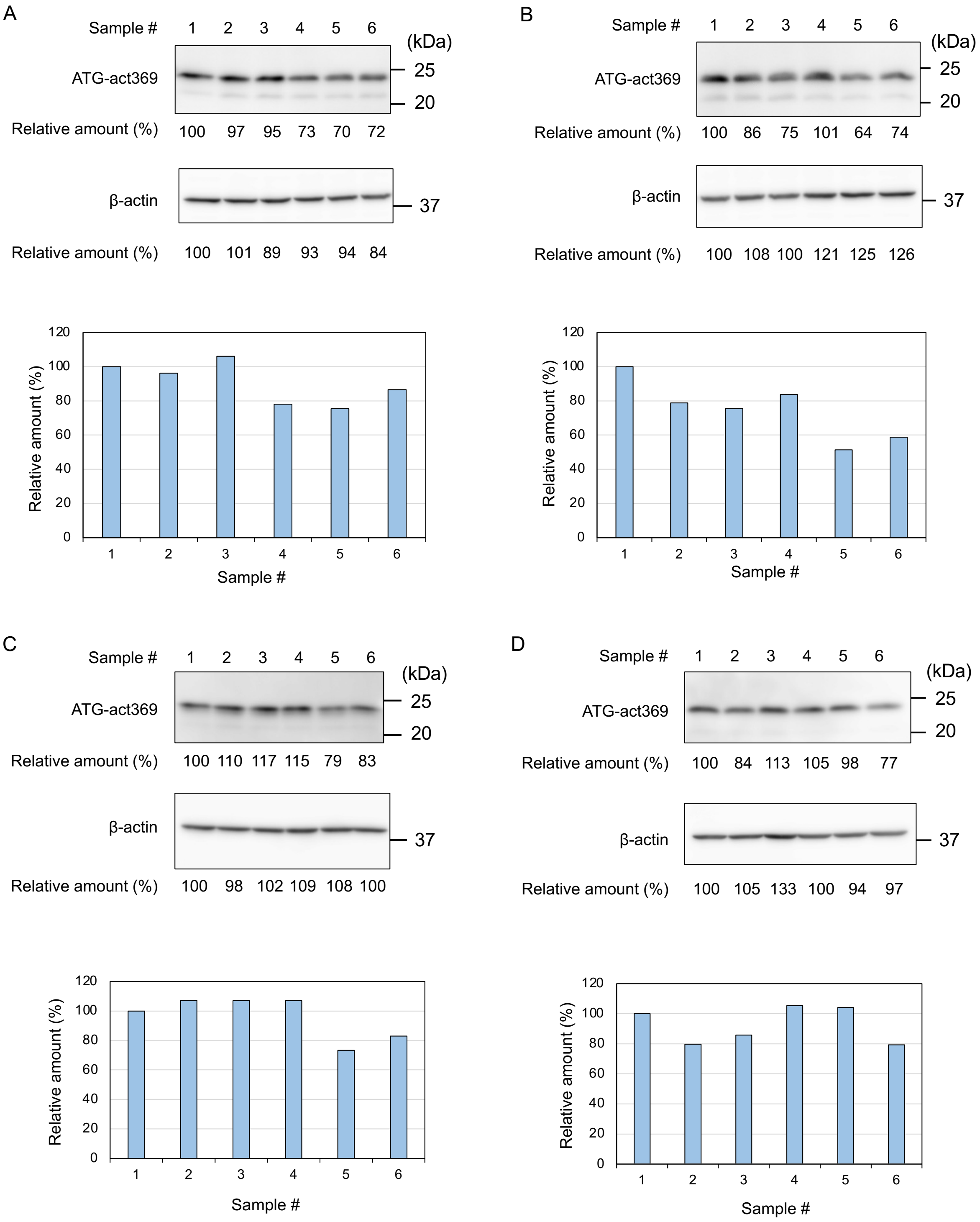


**Figure S7.** Individual data for Figure 4C.

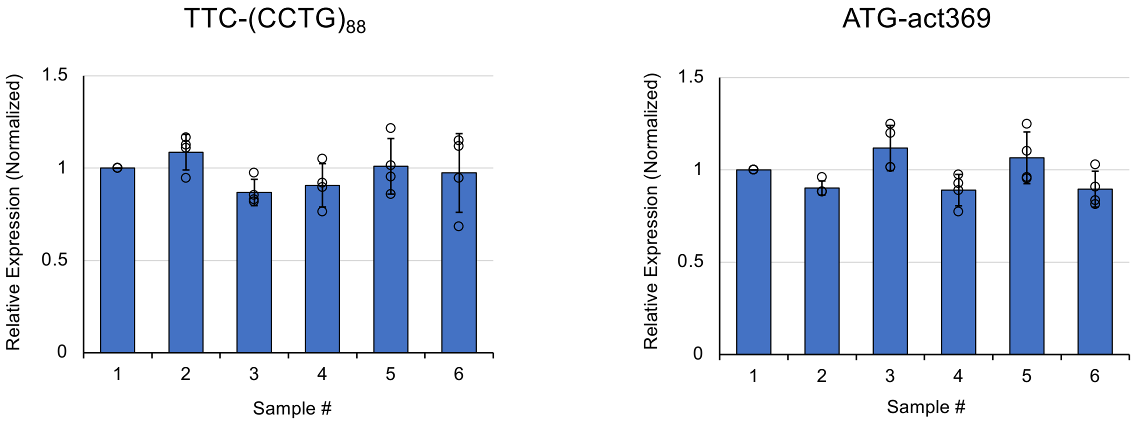


**Figure S8.** Relative RNA levels from the transfected plasmid containing TTC-$(CCTG)_{88}$ or ATG-act369. Relative RNA levels were calculated using the $2^{-\Delta\Delta Ct}$ method with the endogenous actin expression level as the normalizing control [Livak and Schmittgen (2001) *Methods* 25, 402-408. DOI: 10.1006/meth.2001.1262]. Values are presented as means ±SD (n=4). Each data point is represented by an open circle.

**Method**

***RT-qPCR***

Total RNA from cells was prepared using NucleoSpin RNA Plus (Macherey Nagel). RT-qPCR for RNA transcribed from plasmids was performed with One Step TB Green PrimeScript PLUS RT-PCR Kit (Takara) and detected using the LightCycler 96 System (Roche). RT-qPCR for endogenous actin was performed as a control for normalization. The sequences of each primer pair were (5'-3') :

*For plasmids: GGATCCGGCTCGAGGACTAC and GGCACGTCGTAAGGGTAGCTAC*

(see also Fig. S1)

*For endogenous actin: ATGACCCAGATCATGTTTGAGACC and CACGATGCCAGTGGTACGG*

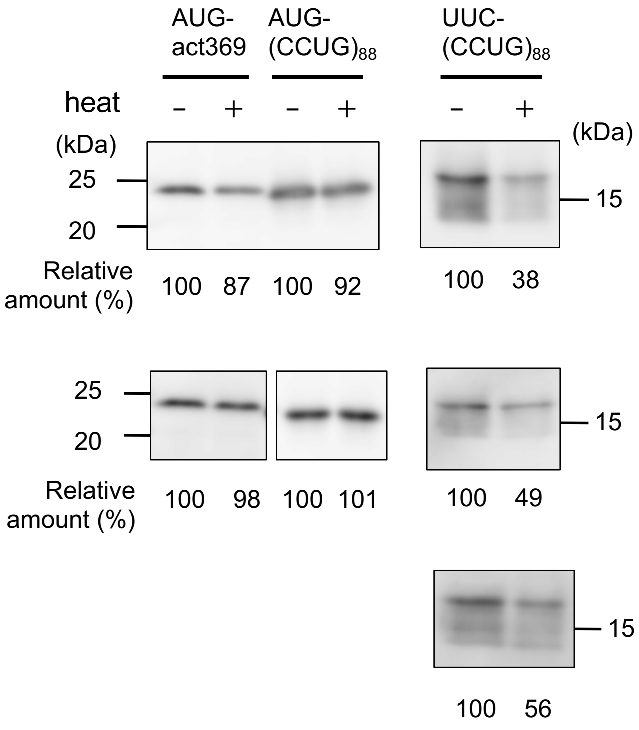


**Figure S9.** Additional independent replicates were performed for Figure 5.

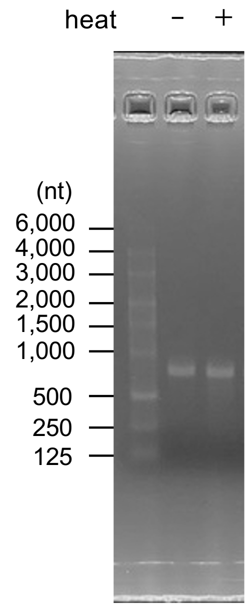


**Figure S10.** Gel-check of UUC-$(CCUG)_{88}$ RNA treated with (+) or without (–) heat. These RNAs were used for translation in the CFPS system in Figure 5.

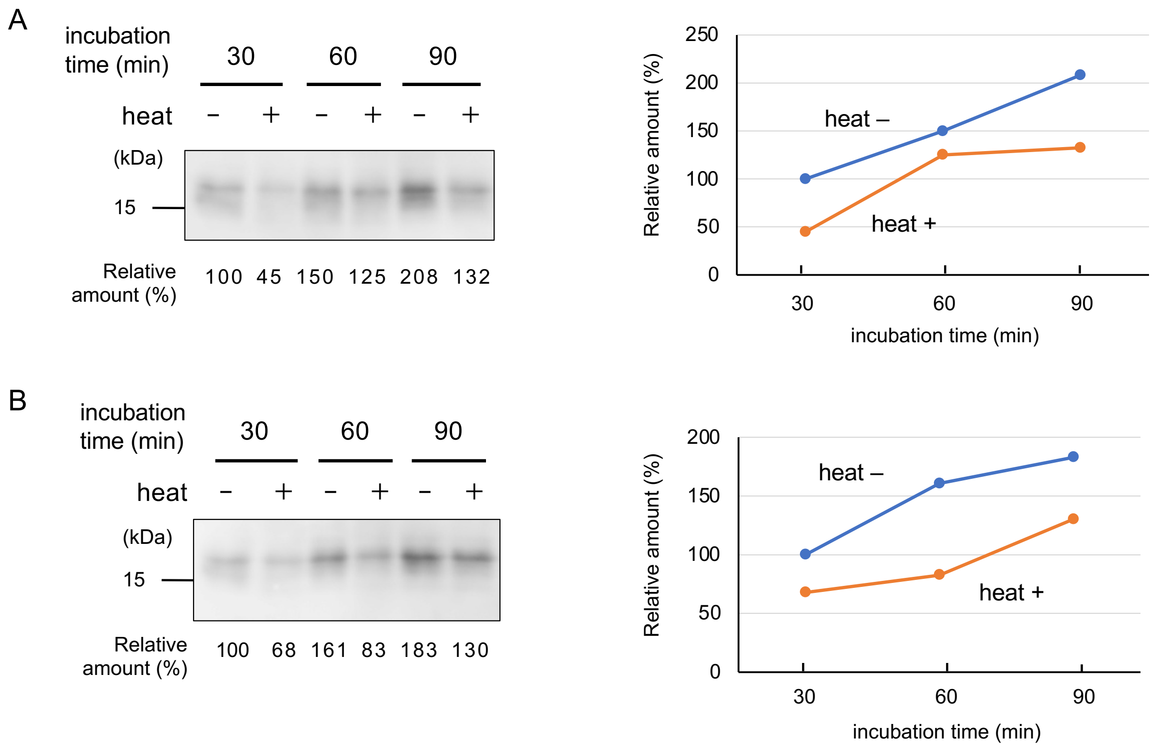


**Figure S11.** Time course experiments of RAN translation from heat-denatured (heat +) or non-denatured (heat –) CCUG-repeat RNA in the CFPS system. Two independent replicates were performed (A and B). A portion of the heat-denatured UUC-$(CCUG)_{88}$ RNA seems to be reverted to a native state during incubation.

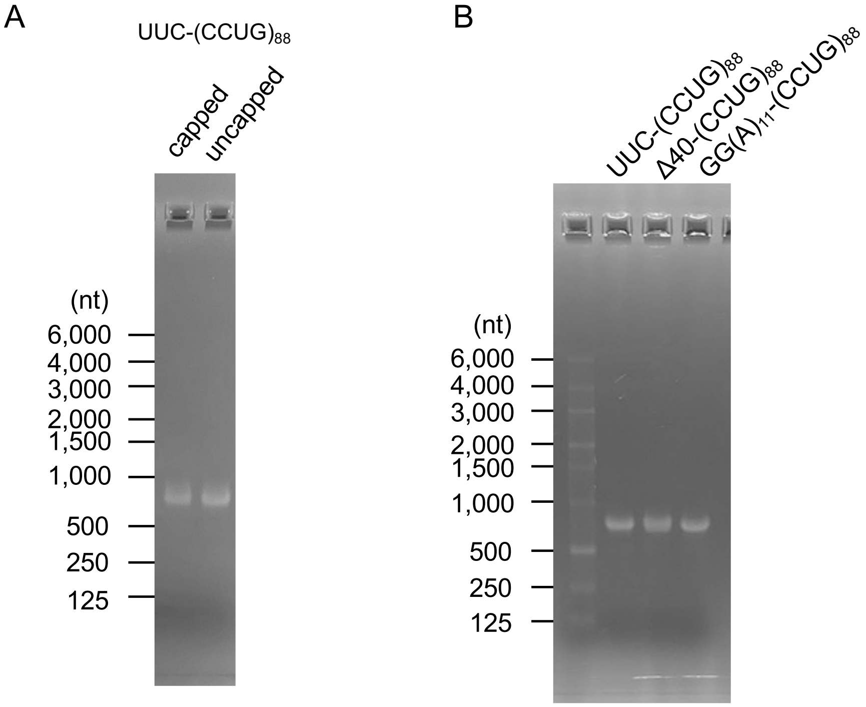


**Figure S12.** Gel-check of *in vitro* transcribed RNAs for Figure 7. (A) RNAs of capped or uncapped UUC-$(CCUG)_{88}$ used for the translation in the CFPS system in Figure 7A. (B) RNAs of UUC-$(CCUG)_{88}$, Δ40-$(CCUG)_{88}$, and GG$(A)_{11}$-$(CCUG)_{88}$ used for the translation in the CFPS system in Figure 7B.

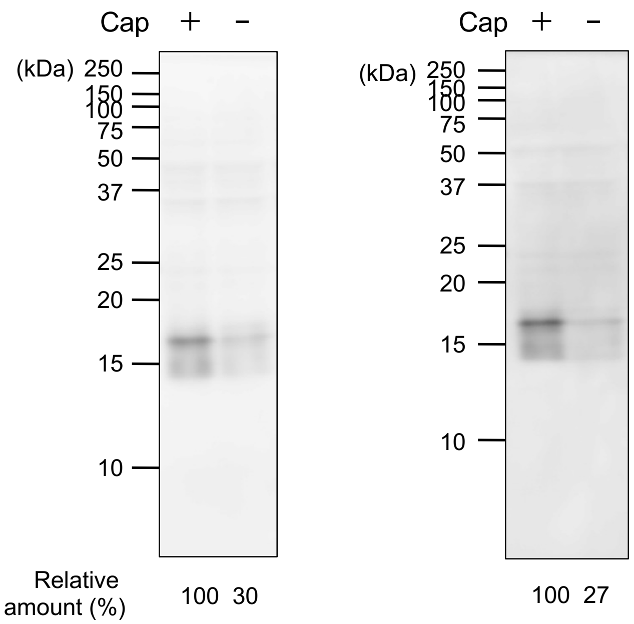


**Figure S13.** Two additional independent replicates were performed for Figure 7A.

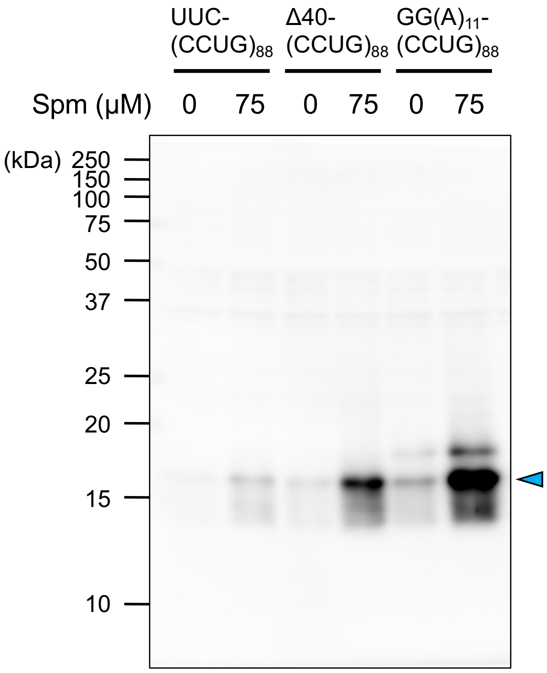


**Figure S14.** Another same experiment was performed for Figure 7B.

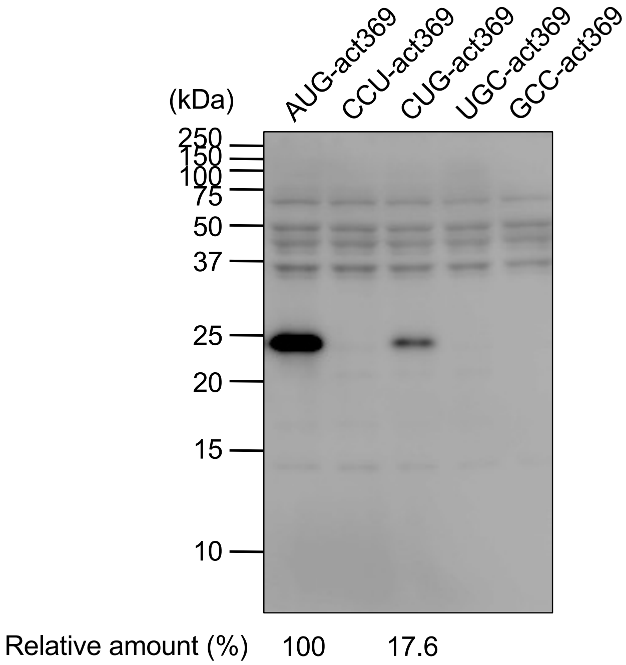


**Figure S15.** Translation products from AUG-act369, CCU-act369, CUG-act369, UGC-act369, and GCC-act369 RNAs in the CFPS system were detected by Western blotting with the anti-HA antibody. Relative amounts of the translation products from AUG- and CUG-act369 were calculated from the intensity of each protein band with the AUG-act369 control set to 100%.

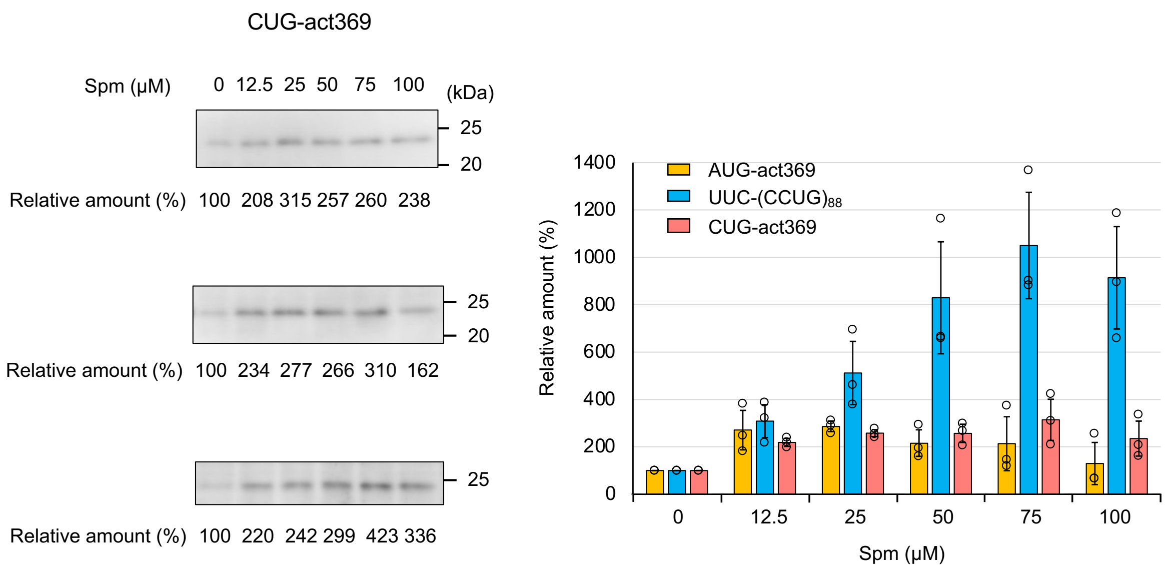


**Figure S16.** Translation products from CUG-act369 RNA in the CFPS system with Spm were detected by Western blotting with the anti-HA antibody. Relative amounts of the translation products were calculated from the intensity of each protein band with the 0 μM Spm control set to 100%. The graph data for AUG-act369 and UUC-$(CCUG)_{88}$ are from Figure 3C. Each data point is represented by an open circle.

**Table S1**. Binding of Spm to RNA

| | Spm (pmol /µg of RNA) | | | | |
|---|---|---|---|---|---|
| RNA | Sample #1 | Sample #2 | Sample #3 | Average | SD |
| $(CCUG)_{88}$ | 3.1 | 4.2 | 1.8 | 3.0 | 1.2 |
| act369 | 13.9 | 4.7 | 3.0 | 7.2 | 5.9 |
| no RNA | <0.1 | <0.1 | <0.1 | - | - |

**Method**

RNA-polyamine complexes were purified as follows. Ten µg of UUC-$(CCUG)_{88}$ or CUG-act369 mRNA (for a negative control, no RNA was added) was incubated in the HeLa CFPS system (10 µl) in the presence of 0.75 mM Spm for 30 min at 32°C. After addition of a buffer (10 µl; 1 M NaCl, 2 mM EDTA, 10 mM DTT), the sample was mixed with the Oligo $d(T)_{25}$ Magnetic Beads (20 µl; NEB: S1419S) equilibrated with a buffer (20 mM HEPES-KOH pH7.5, 500 mM NaCl, 1 mM EDTA, 5 mM DTT). Following incubation for 30 min at 22°C, the sample tube was placed into the magnetic rack so that the magnetic beads were pulled to one side of the tube, and then the supernatant was removed. For the 1$^{st}$ wash, the magnetic beads were mixed with of a buffer (20 µl; 20 mM HEPES-KOH pH7.5, 500 mM NaCl, 1 mM EDTA), and supernatant was removed after incubation for 2 min at 22°C. For the 2$^{nd}$ wash, a lower-salt buffer (20 µl; 20 mM HEPES-KOH pH7.5, 200 mM NaCl, 1 mM EDTA) was used. For elution of RNA, a buffer (20 µl; 20 mM HEPES-KOH pH 7.5, 1 mM EDTA) was added to the beads, and the tube was vortexed gently. After incubation for 5 min at 50°C, the eluate (20 µl) was obtained. The eluates were treated with trichloroacetic acid (5%, final concentration) for 30 min at 90°C and cooled on ice for 5 min. After centrifuging twice at 21,600 × *g* for 15 min each, the supernatants were obtained for the analysis using a Jasco HPLC system with an InertSustain C18 column (GL Science). Samples #1-3: three independent experiments.
